# Risk Factors for Recurrence of Primary Sclerosing Cholangitis after Liver Transplantation: Single-Center Data

**DOI:** 10.3390/jpm14030222

**Published:** 2024-02-20

**Authors:** Elisa Catanzaro, Enrico Gringeri, Nora Cazzagon, Annarosa Floreani, Umberto Cillo, Patrizia Burra, Martina Gambato

**Affiliations:** 1 Multivisceral Transplant Unit and Gastroenterology, Department of Surgery, Oncology, and Gastroenterology, Padova University Hospital, 35128 Padova, Italy; 2 Hepatobiliary Surgery and Liver Transplantation Center, Department of Surgery, Oncology, and Gastroenterology, Padova University Hospital, 35128 Padova, Italy; 3 Scientific Institute for Research, Hospitalization and Healthcare, 37024 Verona, Italy; 4 Department of Surgery, Oncology, and Gastroenterology, Padova University Hospital, 35128 Padova, Italy

**Keywords:** liver transplantation, primary sclerosing cholangitis, recurrence, cholestatic disease, biliary strictures, inflammatory bowel disease, risk factors

## Abstract

Background: Primary sclerosing cholangitis (PSC), comprising 5–15% of European liver transplantation (LT) cases, poses a significant challenge due to the risk of post-transplant disease recurrence (rPSC). This single-center study aimed to determine the rPSC rate and long-term post-LT outcomes in PSC patients and to identify potentially modifiable risk factors of rPSC. Methods: All PSC patients receiving LT at Padua Hospital from 1993 to 2021 were included. Recipient data were collected pre-LT, at LT, and during the follow-up. Donor and LT features were recorded. The rPSC rate was assessed according to Mayo Clinic criteria. Patient and graft survival were reported. Results: Thirty-three patients were included. The main indication of LT was decompensated cirrhosis (70%). Nine patients (27%) developed rPSC during a median follow-up of 59 months (45–72). A longer cold ischemia time (*p* = 0.026), donor female gender (*p* = 0.049), inflammatory bowel disease reactivation (IBD) post LT (*p* = 0.005) and hepaticojejunostomy (*p* = 0.019) were associated with a higher risk of rPSC. Graft and patient survival at 1, 5 and 10 years post LT, 94%, 86%, 74% and 97%, 89%, 77% respectively, were not affected by rPSC development. Conclusion: Specific donor and surgical features might increase the risk of rPSC. Identifying predictive factors for rPSC to prevent graft loss is challenging but could lead to a more personalized organ allocation and follow-up in PSC transplanted patients. IBD reactivation might have a pathogenic role in rPSC. In our single-center experience, rPSC did not affect patient and graft survival.

## 1. Introduction

Primary sclerosing cholangitis (PSC) is a rare, cholestatic disorder characterized by chronic intra- and extrahepatic bile ducts inflammation, progressing to biliary strictures and dilatations. PSC prevalently affects 30–40-year-old males, more commonly in northern countries, with an incidence range approximately from 0.5 to 1.3 cases per 100,000 person-years [1]. It is associated with inflammatory bowel disease (IBD) in up to 80% of patients, with ulcerative colitis (UC) representing the most prevalent phenotype (80%) [2,3,4]. PSC is characterized by a slow evolutive progression with chronic inflammation involving liver parenchyma, leading to liver fibrosis and cirrhosis with portal hypertension over a 10–15-year period [5,6,7]. PSC is highly correlated with the risk of hepatobiliary malignancies, mainly cholangiocarcinoma (CCA) as well as colorectal cancer (CRC), generally when IBD is present [8]. The pathogenesis of PSC remains unknown. It is considered an immune-mediated condition, and different pathogenic hypothesis have been proposed, involving bile acid toxicity and gut bacterial translocation in the biliary tract [9,10,11]. Currently, liver transplantation (LT) represents the only curative option for PSC patients; recurrent cholangitis, intractable pruritus and developing PSC-related perihilar CCA represent LT indications for PSC [12,13]. The main concern after LT is the recurrence of PSC (rPSC), which is diagnosed based on the combination of radiological and histological features, with evidence of diffuse non-anastomotic biliary lesions that occur in the absence of vascular complications [14]. The recurrent PSC rate is reported in up to 25% of transplanted patients with a negative impact on graft survival and need of re-transplantation (reLT) [15,16]. Recurrent cholangitis or concomitant IBD are shown to play a role in the development of biliary damage and rPSC after LT [17,18]. Several risk factors for the development of rPSC have been reported so far, including cellular rejection or immunosuppressant treatment, as well as surgical transplant techniques, but their role is still uncertain [19].

The aim of this study was to assess PSC recurrence rate and the related risk factors in a single transplant center and the impact of rPSC on the long-term outcomes after LT. The identification of modifiable risk factors for rPSC is focused on defining strategies that could potentially reduce the rate of recurrence in transplanted patients.

## 2. Materials and Methods

We analyzed data from all consecutive LTs performed for PSC between 1993 and 2021 at Padua Liver Transplant Center. The follow-up was until April 2022. Patients with variant syndromes (with features of autoimmune hepatitis [AIH] or primary biliary cholangitis [PBC]) were also included. Th exclusion criteria were patients from the Pediatric Unit, the presence of secondary causes of sclerosing cholangitis, including immunoglobulin G4 (IgG4)-related cholangiopathy, bacterial, viral, and parasitic infection, or iatrogenic cholangiopathy.

The diagnosis of PSC was defined according to the European Association for the Study of the Liver (EASL) guidelines: the presence of typical cholangiographic features in the biliary tree and/or histological diagnosis (histology was mandatory in small duct PSC or overlap syndromes) [20].

Demographic and clinical recipient data were collected before LT, at the time of LT and during the follow-up. The following variables were recorded: demographics (age, gender), variant syndromes, model of end-stage liver disease (MELD) at the time of LT and indication for LT, immunosuppression regimen, including steroid therapy duration (defining >6-months as “maintenance therapy”), episodes of rejection, and any diagnosis of hepatobiliary neoplasms or CRC. The following donor and LT features were collected: gender, age, cold ischemia time (CIT), and type of transplantation and biliary reconstruction. In patients with IBD, the following data were recorded: date of diagnosis, IBD phenotype, treatment before LT (5-aminosalicylic acid/mesalazine, steroids, immunosuppressive therapies, biologic drugs), history of bowel surgery or colectomy, and IBD activity after LT. The reactivation of IBD was defined by the presence of endoscopic and histological reactivation, as shown in Table 1. Moderate and severe disease were classified according to the Mayo Endoscopic Score (MES) and Simple Endoscopic Score—Crohn’s Disease disease (SES-CD) [21].

The primary outcome was PSC recurrence, diagnosed according to the Mayo criteria from Graziadei et al., including the following: a confirmed diagnosis of PSC before LT; intra- and/or extrahepatic biliary strictures, beading, and irregularities occurring on cholangiograms > 90 days after LT; and histological findings of fibrous cholangitis and/or fibro-obliterative lesions, with or without ductopenia, and biliary fibrosis or cirrhosis [14]. Clinical features of rPSC were collected, including the date of diagnosis, symptoms at the time of diagnosis, and the development of cirrhosis or recurrent cholangitis after diagnosis. Secondary outcomes were patient and graft survival. Any cause of death was registered, both liver and non-liver related.

All data were analyzed using the statistical package SPSS (version 26.0; SPSS, Inc., Chicago, IL, USA). Quantitative variables were expressed as mean values ±1 standard deviation and/or median values (range) if their distribution was skewed. Fisher’s test was used for categorical variables, and the Mann–Whitney U test was used for the comparison of quantitative variables. Results with a two-sided *p*-value ≤ 0.05 were considered statistically significant. Both patient and graft survival were analyzed using the Kaplan–Meier method. The study has been conducted according to the ethical guidelines of the 2013 Declaration of Helsinki and approved by the Local Ethics Committee. Informed consent was obtained according to our center protocol. 

## 3. Results

### 3.1. Patient Population

Between 1993 and 2021, 1917 patients underwent LT at Padua Liver Transplant Center. In 44 patients (2%), the indication for LT was PSC. The number of LT for PSC increased progressively during the study period (1993–1999, *n* = 4; 2000–2007, *n* = 13; 2008–2014, *n* = 8; 2015–2021 = 21), reaching 9% of all LT indications in 2021.

**Figure 1:**
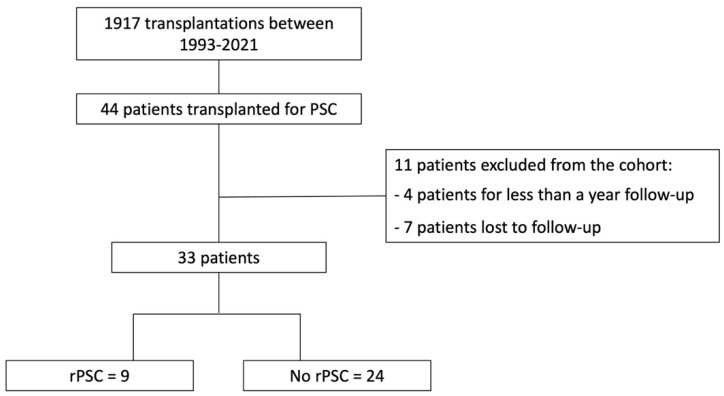
Flowchart of the study.

In the final analysis, as reported in Figure 1, 33 patients with PSC underwent LT, nearly equally gender-distributed, with a median age at transplant of 44 years. 

Baseline demographic and clinical features are summarized in Table 1. 

Among all PSC patients, eight patients (24%) were diagnosed with a variant syndrome; in seven patients (88%), it was an AIH variant syndrome, and one patient (12%) had a PBC variant syndrome. The autoimmune comorbidities observed were systemic lupus erythematosus (SLE) (6%), Hashimoto’s hypothyroidism (3%), hyperthyroidism (3%), psoriasis (3%), and pemphigus (3%). All patients received ursodeoxycholic acid (UDCA) before LT, in accordance with PSC therapy.

### 3.2. Transplant Features

Most PSC patients were listed due to end-stage liver disease, and 23 patients (70%) were transplanted for acute decompensation. Other indications were the presence of recurrent cholangitis in nine patients (27%) and pCCA in one patient (3%), according to the Mayo Clinic protocol [22]. The median MELD score was 17 (IQR 10.5–26.0) (available in 26 patients). The median donor age was 52 years (IQR 26.5–68.6), the majority were male (53.1%), and all donation after brain death (DBD). Mismatch between donor and recipient gender occurred in 13 cases (40.6%). The median cold ischemia time was 8 h (IQR 6.3–9). A full-size graft was used in 29 patients (88%), and a split graft LT was performed in only 4 patients (12%). Roux-en-Y hepaticojejunostomy and T-T anastomosis were performed in 14 (42%) and 19 (58%) patients, respectively. Donor and recipient features are shown in Table 1.

The main immunosuppressant used was tacrolimus (88%) in association with mycophenolate mofetil (MMF) in most of patients (57%). Data on steroid therapy were available in 28 patients, and all of them received steroid therapy as the third drug for less than 6 months in most patients (64%).

### 3.3. PSC Recurrence after LT

**Figure 2:**
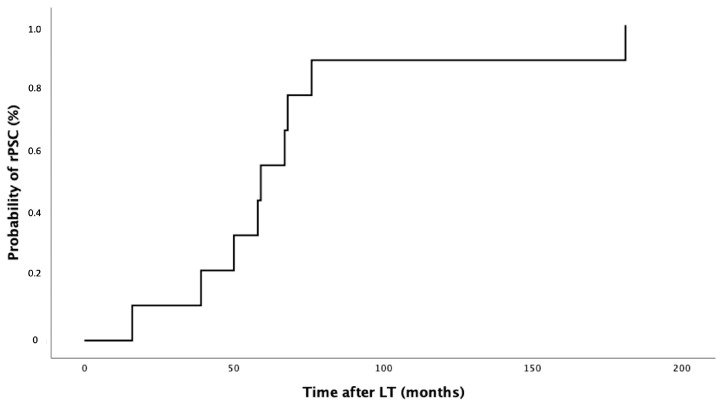
Probability of rPSC in the study cohort.

After LT, nine patients (27%) developed PSC recurrence with a median follow-up of 59 months (range 45.5–72.0), as shown in Figure 2. In most patients (*n* = 5; 55%), rPSC occurred within 5 years after LT, in three patients (33%), between 5 and 10 years after LT, and in one patient (11%), more than 10 years after LT. Eight out of nine patients (89%) with rPSC developed symptomatic recurrence with multiple episodes of cholangitis. One-third of patients (*n* = 3, 33%) developed advanced liver disease in a median time of 30 months after LT.

### 3.4. Patient and Graft Survival

**Figure 3:**
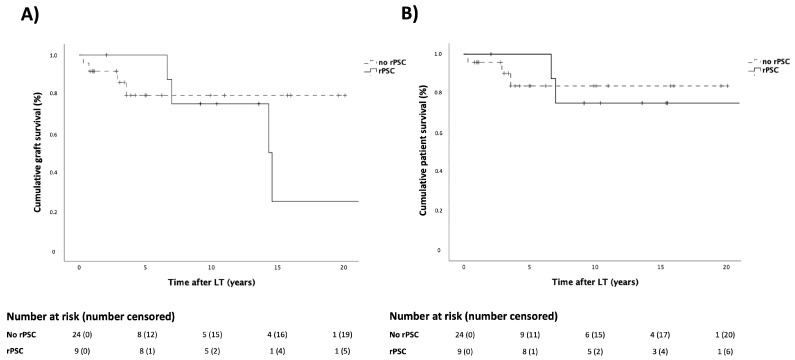
Graft (**A**) and patient (**B**) survival according to rPSC development.

In the overall population of PSC patients, graft survival at 1, 5 and 10 years after LT was 94%, 86% and 74%, respectively. The graft survival rate in the rPSC group at 1, 5, and 10 years after LT was found to be similar to that of the no-rPSC group (100% vs. 92%, 100% vs. 79%, and 75% vs. 79%, respectively; *p* = 0.524), as shown in Figure 3A. Six patients were listed for re-LT with the following indications: recurrence of PSC (*n* = 4; 66%), primary non-function (*n* = 1; 17%), and late vascular complications (*n* = 1; 17%). Half of the rPSC patients (two out of four) underwent reLT, with recurrent cholangitis serving as the indication for reLT. In the overall population of PSC patients, patient survival at 1, 5, and 10 years after LT was 97%, 89%, and 77%. The patient survival rate in the rPSC group at 1, 5, and 10 years after LT was found to be similar to that of the no-rPSC group (100% vs. 96%, 100% vs. 84%, and 75% vs. 84%, respectively; *p* = 0.992), as shown in Figure 3B. Causes of death were liver-related in four out of five patients (80%).

In our cohort, we registered the incidental presence of intrahepatic CCA in three patients (9%) and HCC in one patient (3%) on explant pathology. Among them, one patient presented CCA recurrence 31 months after LT and died thereafter due to tumor progression.

### 3.5. Recipient, Donor and Transplant Features Risks of rPSC

As shown in Table 1, the presence of cirrhosis (*p* = 1.00) and a history of recurrent cholangitis (*p* = 1.00) were not statistically different between patients who developed rPSC compared to those who did not. A higher median MELD at transplant was observed in those who developed rPSC, 19.5 (10.5–26.0) vs. 17 (9.0–27.0), respectively, without reaching statistical significance (*p* = 0.86). Focusing on donor features, a longer CIT (*p* = 0.03) and donor female gender (*p* = 0.049) were associated with a higher risk of rPSC. Still, data showed an increased rate of rPSC in those patients who received a hepaticojejunostomy (*p* = 0.02). Maintenance steroid therapy did not affect rPSC development (*p* = 0.67). Acute cellular rejection occurred in five patients (15%) with no significant correlation between the episode of acute rejection and recurrence of PSC (*p* = 1.00).

### 3.6. IBD Characteristics

A total of 24 patients (73%) who underwent LT for PSC had a diagnosis of IBD, and most of them had a diagnosis of IBD before LT (79%). The main IBD phenotype was UC (67%). The characteristics of IBD are shown in Table 2. The majority of IBD patients were treated with mesalazine before LT (*n* = 16; 67%). Other associated therapies were azathioprine (AZA) (21%), cyclosporine (4%), or biological therapy (17%). Six patients (18%) did not receive any therapy due to intolerance or low compliance. Three patients (13%) underwent colectomy before LT, due to refractory IBD (in two out of three patients and in one patient for colorectal cancer). A total of 12 patients (50%) presented IBD reactivation after LT. Seven patients (78%) showed recurrence of both diseases (intestinal and liver). All patients with rPSC had a diagnosis of IBD.

As shown in Table 2, the presence of IBD diagnosis, regardless the time of the onset (before or after LT) is associated with a higher risk of developing PSC (*p* = 0.04). Treatment with mesalazine (*p* = 0.71), AZA (*p* = 1.00) or biologic treatment (*p* = 1.00) before LT did not influence recurrence. The reactivation of IBD after LT was significantly associated with a higher risk of developing rPSC (*p* = 0.005).

## 4. Discussion

PSC is still a challenging disease, mostly because both the main pathophysiological mechanisms and consequently the curative therapeutics options are still unclear. In our cohort, we registered an increased number of LT for PSC in the last decade, reaching 9% of all LT in 2021, similar to the rest of Europe, with a reported rate of LT for PSC of up to 10–15% of transplants per year [3]. In the last 20 years, a higher availability of organs due to the increasing use of non-standard donors (non-DBD or older donors) and the increase in PSC diagnosis might be some of the reasons explaining this trend [23]. Reported 1- and 5-year patient survival are satisfying in PSC patients (around 90–97% and 80–85%, respectively) as well as 1- and 5-year graft survival (around 80% and 70%, respectively) comparable to other indications for LT [23,24,25,26,27,28]. Regretfully, the post-LT course is affected by the risk of disease recurrence and consequently graft loss. We registered 27% of rPSC in the long term, similar to previous reports, without a significant impact of rPSC on patient and graft survival [27,29,30]. On the contrary, Visseren et al. reported recently ELTR data, including 1549 transplanted patients, 259 with rPSC, and showing a significant negative impact of rPSC on both graft and patient survival [16]. The small size of previous PSC cohorts, including ours, may explain the underestimated effect of rPSC on survival outcomes.

Several features were associated with higher risk of rPSC, with a wide variability across different studies, still due to the small size of patient populations. We observed an association between rPSC and female donor gender. The role of gender has been already investigated, showing the recipient–donor gender mismatch to be more common in patients with rPSC [31]. In addition, in a metanalysis, a female-to-male mismatch was shown to have a worse impact on PSC patients and graft survival after LT [32]. Different hormone levels and the major susceptibility of female organs to external damage could explain the results, but more studies are needed to validate the specific role of gender in rPSC. In addition, we observed an association between rPSC and a. A previous metanalysis conducted by Stahl et al. already identified an association between prolonged CIT and poor outcomes, including a higher risk of primary non-function after LT and decreased patient and graft survival [33]. Our result could be explained by the possible role of ischemia–reperfusion injury on the graft through a complex interplay of several mechanism including inflammation, cellular edema and necrosis, particularly affecting the biliary tree [31,34]. Ischemia–reperfusion injury seems to affect the contractile structure of bile canaliculus, leading to the development of early intrahepatic cholestasis, associated with the alteration of canalicular microvilli and an increased exposure to toxic bile acids [35]. Furthermore, prolonged CIT was shown to affect bile composition downregulating several Mucin (Muc) expressions, like Muc1, Muc 3A and Muc5B, that may favor biliary insults [36]. All these mechanisms could contribute to the development of biliary strictures after LT, and more interest should be set on the impact of CIT on graft function in this specific setting [37,38].

The impact of different surgical biliary reconstructions on rPSC is still debated. Historically, the Roux-en-Y hepaticojejunostomy technique has been the preferential surgical biliary reconstruction method in patients transplanted for PSC in many LT centers to reduce the risk of disease recurrence and de novo CCA on the remnant biliary tract. However, contrasting results have been reported. Sutton et al. showed a higher rate of non-anastomotic biliary strictures in PSC recipients with Roux-en-Y hepaticojejunostomy compared with duct-to-duct reconstruction [39]. Recently, some studies proved that duct-to-duct anastomosis is associated with lower rates of post-transplant cholangitis and easier endoscopic access, and this type of reconstruction did not affect overall graft outcomes [40,41]. Regarding the risk of de novo CCA, it is extremely low, according to the analysis conducted by Welsh et al., with no difference in the rate of occurrence depending on the type of biliary anastomosis [42]. In our cohort, those patients that underwent LT with a Roux-en-Y hepaticojejunostomy showed higher rPSC prevalence compared with those with duct-to-duct anastomoses. The possible explanation might be related to the higher risk of ascendent shifting of bacteria species coming from the bowel lumen; in fact, Roux-en-Y hepaticojejunostomy provides easier access for bacteria due to the loss of normal continence of the ampulla of Vater. As a confirmation, prompt colonization of hepaticojejunostomy after LT was previously observed, and the higher rate of recurrent ascending cholangitis and intrahepatic colonization seems to correlate with this hypothesis [43,44,45]. Our results suggested a preference for duct-to-duct anastomosis as a surgical technique in LT for PSC. However, the specific biliary tree disease involvement might have an impact on rPSC, and it should be considered in the decision making. Meanwhile, further analysis including larger cohorts and the extrahepatic disease phenotype will be required to confirm this assumption. 

The association of PSC with inflammatory bowel disease is well known, and the IBD prevalence of 73% in our cohort reflects data from the literature [4]. Several factors, such as bowel, liver, and transplant-related features, might influence IBD activity after LT. Nearly one-third of PSC patients after LT might experience a worsening of IBD activity, and this group reached 50% in our cohort [46]. We observed that the presence of IBD and its reactivation was strongly associated with the development of rPSC. Peverelle et al. also observed, in a cohort of 120 patients, that moderate-to-severe bowel activity, identified with elevated levels of inflammation, increased the risk of rPSC with a relative risk of 8.80 [47]. Recently, high interest has been focused on gut microbiota in patients with PSC-IBD, showing unique features. A strong negative impact on mucosal barrier integrity that causes a delay in the translocation of gut microbes and their metabolites has been reported, and the aberrant homing of activated gut-specific lymphocytes in the biliary tract, along the portal vein system, was also observed [48,49]. In addition, mucosal inflammation plays a crucial role in tight junctions permeability, generating the “leaky gut” model that amplifies bacterial translocation and consequentially an inflammatory reaction of the bile ducts [4]. Globally, the impact of active IBD on rPSC is quite settled, but more data are needed to define whether a modification of gut microbiota or preemptive treatment of IBD reactivation might reduce the risk of rPSC.

The identification of risk factors associated with rPSC is an unmet need. In the future, the combination of pre-LT features with surgical or donor factors might help to stratify patients according to their risk of worse outcome after transplantation. This is an important goal to improve quality of life, life expectancy and delaying the onset of rPSC in the PSC population mainly represented by young people. Furthermore, a standardized imaging surveillance protocol for rPSC detection is needed for the early detection of graft dysfunction. 

The simultaneous presence of inflammatory bowel disease (IBD) makes even more complex the risk stratification in the single patient, who presented two different diseases with a close relationship, not fully understood yet. For instance, the role of immunosuppression after LT in the IBD control is not clear. Jørgensen et al. reported a better control of IBD after liver transplant using cyclosporine [50], while recently, Åberg et al. showed higher patient and graft survival in PSC patients immunosuppressed with tacrolimus compared to those who received cyclosporin after LT [51]. More data will be needed to define the best immunosuppression protocol in PSC patients after LT, also considering the risk of IBD reactivation. Lastly, surgical techniques might also have a role on rPSC after LT, as we reported. Therefore, a personalized approach is mandatory to obtain better results in the individual patient profile.

The main limitation of the present study is the small number of patients included and its retrospective nature. However, patient data were fully available, allowing us to characterize accurately the study population and to identify the possible risk factors associated with rPSC. In addition, the diagnosis of rPSC was uniformity made according to the Mayo Clinic criteria.

## 5. Conclusions

In conclusion, this is the first Italian monocentric analysis focused on rPSC characteristics and outcomes. In our center, we showed an rPSC rate of 27% with no impact on patient or graft survival after LT. Some donor features, such as prolonged ischemia time and female donor gender, could expose the graft to a higher risk of developing rPSC, as well as performing a Roux-en-Y biliary reconstruction. Preexisting IBD before LT and a history of IBD reactivation after LT are independent risk factors for rPSC, possibly due to an enhanced bacterial translocation of a specific gut microbiota. A tailored approach regarding organ allocation, patients’ selection, surveillance and immunosuppression protocols after LT in PSC patients should be made with the hopes of providing a significant reduction in rPSC incidence and eventually a better graft and patient outcome. 

## Figures and Tables

**Table 1:** Univariate analysis of recipient and donor potential risk factors for PSC recurrence in our cohort.

Patients, *n* Median (IQR), *n* (%)	Cohort (*n* = 33)	rPSC (*n* = 9)	No rPSC (*n* = 24)	*p*-Value
**Recipient features**				
Recipient gender (male)	16 (48)	5 (56)	12 (50)	1.000
Recipient age (years)	44 (31.3–56.7)	37.0 (27–47.5)	45.0 (31.0–52.8)	0.290
**MELD score at LT**	17 (10.5–26.0) ¶	19.5 (10.5–26.0)	17 (9.0–27.0)	0.866
**Variant syndromes**	8 (24)	3 (33)	5 (21)	0.651
**Cirrhotic evolution**	25 (76)	7 (78)	18 (75)	1.000
**Recurrent cholangitis before LT**	16 (48)	4 (44)	12 (50)	1.000
**Autoimmune overlap**	6 (18)	2 (22)	4 (17)	1.000
**Donor features**				
Donor gender (male)	17 (52) ^§^	2 (22)	15 (65)	0.049
Donor age (years)	52.0 (26.5–68.6) ^§^	55.0 (21.0–67.5)	52.0 (34.0–69.0)	0.773
**Gender mismatch**	13 (41) ^§^	4 (44)	9 (39)	1.000
**Ischemia time (h)**				
CIT	8.0 (6.3–9.0) ^§^	9.1 (8.3–9.5)	7.3 (6.0–8.8)	0.026
**Split graft**	4 (12)	1 (11)	3 (13)	1.000
**Type of anastomosis**				
Roux-en-Y hepaticojejunostomy	14 (42)	7 (78)	7 (29)	0.019
Termino-terminal (T-T) anastomosis	19 (58)	2 (22)	17 (71)	
**Acute rejection**	5 (15)	1 (11)	4 (17)	1.000
**Steroid maintenance therapy**	10 (30)	2 (22)	8 (33)	0.669

¶ Missing data in seven patients; ^§^ missing data in one patient.

**Table 2:** Univariate analysis of IBD features associated with PSC recurrence in our cohort.

Patients, *n* Median (IQR), *n* (%)	Cohort (*n* = 33)	rPSC (*n* = 9)	No rPSC (*n* = 24)	*p*-Value
**Diagnosis of IBD**	24 (73)	9 (100)	15 (63)	0.039
Before LT	21 (88)			
De novo IBD	3 (12)			
**Type of IBD**				
Ulcerative colitis (UC)	16 (67)			
Crohn’s disease (CD)	8 (33)			
**5-aminosalicylic acid treatment before LT**	16 (67)	5 (56)	11 (46)	0.708
**AZA treatment before LT**	5 (21)	1 (11)	4 (17)	1.000
**Biologic treatment before LT**	4 (17)	1 (11)	3 (13)	1.000
**History of IBD reactivation after LT**	12 (50)	7 (78)	5 (21)	0.005

## Data Availability

The data that support the findings of this study are available from the corresponding author upon reasonable request.

## Abbreviations

AIH: autoimmune hepatitis; AZA, azathioprine; CCA, cholangiocarcinoma; CD, Crohn’s disease; CIT, cold ischemia time; CRC, colon rectal carcinoma; DBD, donation after brain death; HCC, hepatocellular carcinoma; IBD, inflammatory bowel disease; LT, liver transplantation; MELD, model of end-stage liver disease; MES, Mayo endoscopic score; MMF, mycophenolate mofetil; Muc, mucin; PSC, primary sclerosing cholangitis; reLT, liver retransplantation; rPSC recurrent PSC; SES-CD, Simple Endoscopic Score—Crohn’s Disease; UC, ulcerative colitis; UDCA ursodeoxycholic acid.
